# “Exercise Is My Medicine”: A Qualitative Study Exploring the Experiences of Non-admitted Patients Receiving Physical Activity Promotion From Hospital Surgeons

**DOI:** 10.3389/fpubh.2022.915496

**Published:** 2022-06-03

**Authors:** Stephen Barrett, Stephen Begg, Paul O'Halloran, Kane Rodda, Gabrielle Barrett, Michael Kingsley

**Affiliations:** ^1^Health Promotion Department, Bendigo Health Care Group, Bendigo, VIC, Australia; ^2^La Trobe Rural Health School, La Trobe University, Bendigo, VIC, Australia; ^3^Violet Vines Marshman Centre for Rural Health Research, La Trobe Rural Health School, La Trobe University, Bendigo, VIC, Australia; ^4^Centre for Sport and Social Impact, La Trobe University, Melbourne, VIC, Australia; ^5^Outpatient Rehabilitation Services, Bendigo Health Care Group, Bendigo, VIC, Australia; ^6^Holsworth Research Initiative, La Trobe Rural Health School, La Trobe University, Bendigo, VIC, Australia; ^7^Department of Exercise Sciences, University of Auckland, Auckland, New Zealand

**Keywords:** health promotion, behavior change, motivation, exercise, rural health

## Abstract

**Background:**

Hospital clinicians are increasingly encouraged to use outpatient consultations as an avenue to deliver opportunistic health promotion. There is a dearth of evidence regarding the acceptance of health promotion initiatives from hospital patients themselves.

**Methods:**

We explored the experiences of non-admitted patients who, during a routine consultation with a hospital surgeon received a recommendation to increase physical activity (PA) and a recommendation to engage in a PA telephone coaching program. Twenty-two semi-structured interviews were conducted with individuals who had received the recommendation and proceeded to enroll in a telephone coaching intervention to identify factors that influenced behavior change. Data were analyzed thematically.

**Results:**

Participants' age ranged between 42 and 66 years, with the average age being 54 years. Of the participants, 15 (68%) were women and 7 (32%) were men. Three major themes were identified: (1) the hospital visit represented an opportunity for behavior change that is not to be missed; (2) surgeons were influential in promoting PA change contemplation; and (3) patients welcomed a communication style that promoted autonomy.

**Conclusions:**

Almost all patients considered receiving the recommendation to engage with the telephone coaching as acceptable and helpful toward PA change. Although working in time-restricted consultations, surgeons delivered the recommendation in a patient-centered, autonomy-supportive way, which influenced behavior change. Hospitals should explore avenues to integrate health promotion into routine care, confident of the acceptability and appropriateness of health promotion practice to hospital patients.

## Introduction

Insufficient physical activity (PA) is a major risk factor for chronic diseases, including cardiovascular diseases, type 2 diabetes and some cancers (1, 2). Individuals with chronic disease-related morbidity are frequent users of complex medical care (3). This care is often delivered in non-admitted hospital settings and involves consultations with medical sub-specialities such as endocrinology, renal medicine, general surgery and orthopedic surgery. The management of chronic disease-related morbidity in non-admitted hospital settings accounts for a large proportion of healthcare use and healthcare expenditure (4). Due to the increasing demands of chronic disease management, hospitals have been encouraged to broaden their perspective from curative care to one of more integrated health promotion (5–8).

In order to incorporate health promotion alongside routine care hospitals are encouraged to facilitate patient behavior change by providing interventions to target major risk factors, such as smoking, alcohol, diet and insufficient PA (8). Hospital doctors can play a key role in health promotion by identifying individuals with lifestyle risk factors, such as insufficient PA and utilizing strategies to promote health behavior change (9). These strategies can include having brief conversations around behavior change and/or referring patients to follow-on health promotion services (9, 10). Worldwide, doctors have been encouraged to engage patients in discussions on PA and to make every contact count toward health promotion (9–11).

Despite this encouragement, PA assessment and promotion by hospital doctors is not widespread (12–16), with the absence of referral pathways to specific programs aimed at assisting patients to increase PA being a commonly cited barrier (14–16). Doctors will refer patients to physiotherapy for rehabilitation of specific problems, but report a lack of similar referrals pathways to programs designed to improve PA more generally (14–16). Countries such as Sweden have tried to integrate PA promotion into healthcare delivery through their physical activity on prescription (PAP) model (17). The PAP is a 5-step process that includes consultation, written PA prescription and follow-up (17). The time required to compete these steps has been highlighted as a significant barrier, and doctors would rather refer to other professionals to promote PA change (18). Public Health England examined the feasibility of embedding PA interventions in secondary hospital care, using Sport and Exercise Medicine consultants in the program design (19). Patients were recruited by PA “champions” who were nurses or physiotherapists, not by the consultants. Interviews from participants (*n* = 4) focused on their experience of participating in the behavior change intervention and did not discuss the potential influence of being recruited into the program from the secondary care setting (19).

In the Healthy 4U-2 randomized controlled trial we attempted to overcome the absence of referral pathways to programs designed to improve PA in ambulatory hospital care. Based on the surgeons' preference they were provided with information flyers for a dedicated PA telephone coaching program (16). This enabled them to engage in conversations around potentially increasing PA and provide patients with the flyer and a suggestion that they engage in the telephone coaching service (20). The surgeons felt that this was an achievable way of adding health promotion to routine care given the short amount of time available in consultations (16). Over the recruitment period the surgeons provided 2076 patients with the program flyer and a suggestion to engage with the telephone coaching service. Of these individuals, 33% went on to contact the research team about the telephone coaching program. An exploration of patients' experience of being engaged in PA promotion by surgeons in the non-admitted hospital setting is so far absent from the literature.

Given the high prevalence of chronic disease and the advocacy for hospitals to integrate health promotion into routine care (7, 21), it is important to gain insights from the target audience of these health promotion initiatives, namely hospital patients. Qualitative approaches are useful for examining individual's perceptions and experiences of these processes. The aim of the current study was to explore individuals' experiences of their interactions with the surgeons that included PA discussion and onward referral to a PA telephone coaching program. These insights will provide an in-depth understanding of their experiences, and might offer valuable information to assist with the integration of health promotion into routine hospital practice.

## Methods

This study was a qualitative evaluation that explored the opinions of individuals who were recruited into a telephone coaching intervention study, the results of which have been previously published (20). This current qualitative study was carried out using a qualitative description approach (22, 23). Semi-structured interviews were conducted with a purposeful sample of adults who enrolled into a PA telephone coaching program after receiving a recommendation to engage in the telephone coaching program from a hospital surgeon. Ethical approval was obtained from the research Ethics Committees at Bendigo Health Care Group and La Trobe University College of Science Health and Engineering.

## Sampling and Recruitment

All of the H4U-2 study participants were requested to complete an evaluation form at the 9-month follow-up. The form included a question enquiring whether participants would be willing to participate in a semi-structured interview. Seventy two individuals responded with “yes” and were considered the sample frame for this study. We used a purposive sampling procedure for recruitment to achieve a variation in the participants': (i) change in PA outcomes at the end of the study, measured using accelerometers; (ii) gender, to reflect the sample in the H4U-2 study population; (iii) geographic location (rural or regional); (iv) socio-economic status, using postcodes as a proxy measure; and (v) H4U-2 group allocation (intervention or control). Following completion of the H4U-2 study a research assistant contacted the individuals to confirm their interest in participating. Consent was gained to provide their contact details to the interviewers.

In total, 33 individuals were invited to participate. Following invitation, two people declined to participant; one due to health issues and the other no longer wished to take part. We ceased recruitment once we had our variation sampling requirements and had reached data saturation. In keeping with recommendations we considered data saturation to be when the analysis of additional interviews no longer provided new concepts (24). No new significant information was derived between the twenty-first and twenty-second interview, indicating that data saturation was reached and interviewing was ceased (24).

## Interview Process

We obtained written informed consent from all participants at the start of the interviews. Face-to-face interviews were carried out in the Health Promotion department of the associated hospital between June and September 2020. The first author carried out all interviews. Consistent with the qualitative description approach, we did not adopt any particular theoretical viewpoint a priori (23). We developed an interview guide through team discussion. The interview guide was piloted by interviewing three individuals that received a recommendation to engage with the telephone coaching program by a hospital surgeon and proceeded to take part in a telephone coaching intervention delivered by the Health Promotion department in the hospital. Following these pilot interviews, the final semi-structured interview guide was finalized (Supplementary Material 1). The pilot interviews were not included in the final sample as the individuals were not enrolled into the H4U-2 study (20). Field notes and reflective memos were used to supplement the transcripts to inform the iterative development of interview guides and question-related probes for subsequent interviews.

## Analysis

Data from in-depth interviews were collected and analyzed concurrently. Interviews were audio recorded and transcribed verbatim. NVivo software (Version 12; QSR International, Cambridge, MA, USA) was used to facilitate data analysis. Qualitative description was the analytical approach used for this study. Qualitative description provides straightforward, rich descriptions of experiences or events in a language similar to the participant's own (22, 23). According to Sandelowski, qualitative description is the “least interpretive of the qualitative analysis approaches”, and encourages representing the data as close to the individuals' terms as possible (22). In keeping with this approach, codes were derived from data rather than being determined beforehand and a coding scheme was applied to the interview text. The coded text was then grouped into more general categories, which were reviewed by the research team and merged into themes to help understand the experiences of non-admitted patients received a recommendation to engage with the telephone coaching program by a hospital surgeon. The coding framework was developed and independently trialed on 20% of the transcripts by authors (SBa and KR). Subsequent interviews were independently analyzed by two researchers. Where new data emerged from the interviews the coding frame was refined. A single team member synthesized the findings from all of the team member's coding analysis. The synthesized findings were discussed by the research team with a high level of consensus reached amongst the team.

Descriptive validity was ensured through the accurate recounting of the events and experiences as described by the participants (25). We addressed interpretive validity during interviews through the use of iterative questions and probes that sought to clarify responses (25). This permitted the attainment of sufficiently rich data that accurately depicted the participants' experiences. Credibility and trustworthiness was ensured through data triangulation (interviews, field notes and reflective memos), independent dual coding of transcripts, team consensus on thematic development and the use of verbatim quotations (26).

## Results

Twenty two people participated in semi-structured interviews, where 15 (68%) were women and 7 (32%) were men. The average age of participants was 54 (± 5) years, with participants ranging in age from 42 to 66 years. Table 1 provides details of the participants' characteristics. All participants had completed the H4U-2 study when the interviews took place. The interviews ranged in duration from 26 to 47 min, with an average duration of 35 min.

**Table 1:** Profile characteristics of participants (*N* = 22).

**Marital Status**	
Married/living together	18
Widowed	1
Single	3
**Highest completed education**	
Secondary/high school	6
Post-school vocational	10
University	6
**Employment**	
Working full-time	15
Working part-time	4
Retired	3
**Geographic location**	
Regional	14
Rural	8
**Socioeconomic area^a^**	
1	5
2	4
3	5
4	5
5	3
**Physical activity level at end of intervention^b^**	
Meets guidelines	15
Does not meet guidelines	7
**Pattern of physical activity from baseline to final measurement^b^**	
Increased	15
No change	5
Decreased	2

a *Index of Relative Socio-economic Disadvantage (IRSD) SEIFA scores. IRSD data is presented as quintiles where 1 represents most disadvantaged, and 5 represents least disadvantaged*.

b *Physical activity measurements were taken from accelerometer data as part of the Healthy 4U-2 randomized controlled trial*.

We developed 85 codes, which were applied 388 times to 423 excerpts of text. The number of new codes that were generated decreased with time; interviews 20, 21 and 22 produced four, one and zero new codes, respectively. Given the low numbers of new codes generated over time, and that no new codes emerged from the twenty-second interview, additional interviewing was ceased. Three themes were identified from the analysis: (1) hospital appointments represent an opportunity not to be missed; (2) the influence of the surgeon on behavior change contemplation; and (3) a communication style that promoted autonomy. The codes and categories of the corresponding themes are detailed in Table 2. These themes are described in detail below using verbatim quotes from participants to illustrate and substantiate the themes.

**Table 2:** Codes, categories and themes of patients' experience of being engaged in PA promotion by surgeons.

**Codes**	**Categories**	**Themes**
• Behaviors that have led to hospital appointment • How to improve health • Fork in the road moment • What does the future hold? • Moments of contemplation • Concerns about health • Role of physical activity in health • Turning point • Opportunity to change • Stress of hospital visit • Chance to take perspective on behaviors	° Coming to hospital influenced change ° Significant event coming to hospital clinic ° Promoted thoughts about becoming more active ° Didn't expect conversations on physical activity but appreciated them	Hospital appointments represent an opportunity not to be missed
• Reputation of surgeon • Strong influence on change • Raised awareness of need to change • Short conversation but powerful • Esteemed medical advice • Desire to do the right thing • Could sense the importance in physical activity • Value the surgeon having this conversation • Busy clinicians; realize that topic is important • Want to please surgeon	° Influential professional ° It stressed the importance of change ° Should listen to this advice ° If surgeons makes time for this conversation it must be important	The influence of the surgeon on health behavior change
• Respectful of choice • Sense of control over outcome • Raised awareness of why to change, but it was still my choice • Laid out facts in respectful way • Always a choice • Gave room to consider options • Demonstrated importance of PA, but didn't force the referral	° Felt listened to ° Knew it was important, but I had choice over decision ° Non-judgemental style ° Directive toward change, but not forced	Communication style that promoted autonomy

## Hospital Appointments Represent an Opportunity Not to be Missed

Many participants did not expect to be engaged in discussion around PA during the non-admitted hospital appointment. Most had anticipated a very transactional model of care consisting of a brief consultation with the surgeon specific to the presenting condition. Participants were very happy to have received the health promotion intervention by the surgeon, and considered hospital appointments as a favorable setting in which to promote health behavior change.

*I know you are not going into hospital, like to stay. But there still is a bit of a feeling that, ‘I am going to hospital for an appointment. How did I get here?' So I think there is something in that. I don't know if it's a vulnerability. But there is definitely an opportunity. (Female, 54)*.

Many participants reported that the hospital appointment was a noteworthy event in their life, and they attached a significance to that event. Being referred for a hospital appointment triggered thoughts around lifestyle choices for many and might have initiated the process of contemplating behavior change.

*How many times do you see your GP every year, two, three, four maybe? How often do you get referred to see a surgeon? So it's big… it's a big deal. You start thinking about why am I coming here, could I have done anything about it? I think it really gets you thinking about the choices you make. (Male, 50)*.

*I know for me there was a certain stress in the build-up to the appointment, the days before it and all. Had me thinking about things, looking at myself. And when you go to the hospital and someone presents you with an opportunity to change, well then you might be ready to take it. Being sent to hospital can sure bring you down to earth. (Female, 51)*.

## The Influence of the Surgeon on Health Behavior Change

Participants described the strong influence that the brief interaction with the surgeon had on their decision to contemplate increasing PA and in enrolling into the telephone coaching program. Surgeons were perceived as credible sources of information and the importance of the message to consider increasing PA was amplified as a result of their medical status.

*When a surgeons sits you down and gets you to take a good look at yourself, then it sinks in. You start to think, what road am I really on here? (Male, 52)*.

According to many patients, surgeons had a presence, often described as a voice of authority. As a result, health promotion messages from surgeons were perceived as carrying more weight than messages from other clinicians, including general practitioners.

*To me, maybe it's my age but there is still a sort of an aura around the surgeon. You know, they are a cut above the GP for me. They hold a bit of power, and a bit of influence. So when they tell you to do something, you are probably more likely to do it; you might think “this persons knows what they are talking about”. (Female, 48)*.

*I think even though you spend little time with them they have a sort of an influence. They are a bit different, a change from the GP… can maybe have more influence on decisions. (Female, 61)*.

Receiving the recommendation to engage in the telephone coaching from the surgeon strengthened the significance of the health message and the importance of behavior change. Participants appreciated that surgeons work under time constraints and that they need to prioritize time toward discussing the presenting condition. The fact that surgeons were willing to commit valuable time to engage in discussions on increasing PA seemed to resonate with participants. This heightened the value that surgeons placed on this behavior and as a result participants were increasingly likely to consider behavior change.

*They (surgeons) have a reputation right. And they are busy, and need to be focusing on their job, which is the surgery bit. If they are willing to take time out to talk about getting fitter then you start to think ‘I might need to listen here'. This might actually be important. And I think you take that home with you. (Female, 46)*.

*So then the surgeon has you at a bit of a weak moment I think, you are in the palm of his hands, because really, you are helpless – if he says you need the op, you need the op. So some of it is beyond your control. So when he talks about exercise, maybe your ears perk up. Because now it's kinda (sic), medical advice, to get fit. And that might be powerful. (Male, 53)*.

## Communication Style That Promoted Autonomy

For many of the participants the way the surgeons delivered information about PA behavior change was important in terms of the acceptability of the message and the subsequent uptake. A non-judgemental style that centered on the individual making the choice provided a strong platform from which to base discussions on behavior change.

*He was on the lines of ‘I can't do it for you, you are gonna have to do it for yourself' kinda (sic) business. And I thought ‘Okay, he's made a bit of a point of this exercise thing, so maybe I'll give it a bit of a go'. (Female, 55)*.

*That conversation for me was when I was at the fork in the road. They didn't tell me which road to take, but the conversation really put me on the right road. It got me moving, and then exercising. I can't imagine myself not exercising now, for me, exercise is my medicine. And I think it started there. (Female, 46)*.

Specific components within the delivery of the PA recommendation were consistently highlighted. The surgeons were able to engage patients in conversations around PA behavior change and provide information about a dedicated behavior change program. Participants did not raise any issues with having these conversations and they were appreciative that the engagement with the follow-on behavior change program was a recommendation and not an unsolicited referral. Participants felt like they were part of the conversation and not being dictated to.

*He didn't say you must do this, I'm still there with a decision to make. But now, the consideration I had [making behaviors change] for some time became more important. And, perhaps as important, is this doctor had given me the piece of paper (information flyer). He's kinda (sic) said ‘you need to get yourself in gear, and these guys will help you get there'. (Female, 50)*.

*So, I'd say he wasn't judging me, it was about taking care of myself. I think that's maybe a way to consider it actually, he wanted me to take better care of myself, for my health. But, I didn't feel like the surgeon was telling me what to do. He had a look at me, and my history and probably said, ‘hey, you need to get yourself in gear' and pointed me into the right direction. So it's guidance towards what's right, not a push. Because you push me and I'll push right back. (Female, 51)*.

Providing the recommendation to engage in the telephone coaching highlighted to patients the importance of PA behavior change, but leaving the decision to engage in the telephone coaching or not, to the individual was valued by participants. This provided individuals with time to reflect on the conversation and the direction they took from there. Maintaining autonomy over decisions was important to participants, as exemplified by the following quote:

*If the surgeon said to me, ‘you would benefit from exercise, I am going to refer you to a strength training program' then I probably wouldn't have gone. Because that's her opinion on my life, and I never want to join a program. But let me pick, or provide me with an environment that allows me to make my own choices, and look at where I am now. So there are lots of ways to approach it, it just needs to be done smartly. (Female, 54)*.

## Discussion

This study examined the reflections of patients who received a recommendation to engage in telephone coaching from a surgeon in a non-admitted hospital setting and proceeded to enroll into a PA coaching intervention. Three main themes emerged from the analysis. Firstly, from a patient perspective non-admitted hospital appointments represent an important opportunity to deliver health promotion interventions because individuals are willing to accept health promotion interventions in this setting. Secondly, hospital surgeons are perceived as important clinicians to deliver health promotion interventions and can positively influence behavior change contemplation. Finally, the delivery of health promotion messages in a style that promotes partnership and patient autonomy can facilitate the acceptance and uptake of the messages.

Healthcare professionals are being increasingly encouraged to use clinical consultations as an avenue to deliver opportunistic health promotion (e.g., risk factor screening, provision of advice, make referrals) to facilitate behavior change (8–11). Healthcare professionals, including hospital surgeons, believe that not all patients want to receive health promotion information as part of consultations (27–29). As a result, clinicians choose to forego preventive health practice based on misperceptions of patient motivation, and thus miss an important opportunity to promote health behavior change (27, 30). The evidence from the patient perspective across multiple studies counters the clinicians' opinions and shows that patients are willing to accept health promotion interventions in a variety of healthcare settings (31–33). Given patients' willingness to accept preventive health, the low rates of preventive health practice undertaken represent a missed opportunity to provide interventions to those who would benefit (27). To the best of our knowledge, this current study is the first to investigate the perspectives of patients' that, during routine non-admitted care received a recommendation by a surgeon to engage with a telephone coaching program. The patients in our study indicated that the PA recommendation was acceptable and beneficial during clinical interactions with the surgeons. The delivery of PA recommendation in routine consultations provided an opportunity to address prevention alongside the management of presenting health conditions.

Receiving the recommendation to engage with a telephone coaching program by the surgeon was a significant contributing factor toward increasing PA. This was likely shaped by the relationship between the surgeon and the patient (34, 35). In clinical consultations, during the assessment and management of the patient's condition surgeons begin to take majority control of the clinical interaction (34). The requirement for surgery, or not, is predominantly decided by the surgeon who now assumes total control of the treatment pathway (34). The power dynamics between surgeons and patients differ to most other fields of medicine where patients retain some degree of control over treatment (35). As a result, receiving the recommendation to engage with a telephone coaching program from the surgeon magnified the importance of increasing PA and likely influenced the decision to enroll in the telephone coaching program. Indeed, the surgical profession has acknowledged the important role surgeons play in advocating for behavior change contemplation with patients (16). Surgeons considering delivering opportunistic behavior change advice to patients should be encouraged by the high enrolment into PA coaching programs and the acceptance of the PA recommendation.

The participants in our study highlighted that the communication style of the health promotion message was equally important in promoting PA behavior change. Although patients recognized that surgeons can influence change, the surgeons did not demand that patients enroll in the telephone coaching program. The style of interaction in regards to the PA recommendation was described as autonomy supportive (36), where patients had the final decision to engage with the PA coaching service, or not. This was seen as collaborative, no domineering. Surgeons highlighted the importance of PA change and provided the patient with follow-on options, but the patient was required to take the final step toward managing their own health, providing the patient with a sense of empowerment (36, 37). Providing a supportive environment and expressing non-judgemental understanding of patients' needs are consistent with the central tenets of self-determination theory and motivational interviewing (36). This can promote an increased sense of autonomous motivation to engage in PA programs (38). Providing information to patients relative to their needs enhances the acceptability of the message, promotes behavior change contemplation (39, 40) and can increase the likelihood of subsequent behavior change (27). Importantly, communication skills that promote a non-judgemental, autonomy supportive interactions have been shown to increase engagement in behavior change (41), highlighting that while the provision of information is important, how the health information is provided significantly contributed to information uptake (42).

## Implications for Practice

Two of the most commonly cited barriers to preventive health practice are a lack of time to deliver health promotion initiatives, and not having an organizational system to support delivery of behavior change information (27, 43). The time available for clinicians to undertake consultations is unlikely to increase. This should not be taken as a deterrent to integrating preventive health practice as health promotion initiatives can be delivered in a short period of time (31), even as little as 3 min (44, 45). The participants in our study acknowledged that they did not spend a long time in the consultation with the surgeon, but even in that short period the surgeon was able to highlight the importance of increasing PA and provide the relevant information for the follow-on telephone coaching service. Clinicians should not consider behavior change as a binary event, and recognize that intended outcome of integrating health promotion recommendations into routine care is to assist patients move in an optimal way in the spectrum of behavior change contemplation (39, 40).

Having a system in place to support health promotion practice is likely to help integrate preventive health into routine hospital care (33). In a number of reviews, delivery of health promotion interventions increased when clinicians were aware of, and were able to refer patients to follow-on services (46, 47). Keyworth and colleagues' recent review of systematic reviews affirmed this, demonstrating that providing healthcare clinicians with appropriate resources and support facilitates the delivery of health promotion interventions (33). This was exemplified in the Healthy 4U-2 study where surgeons co-designed PA information material for patients and this enabled them to engage patients in discussions on PA change, confident that they had a dedicated program that they could point patients toward to manage their PA needs (20). Hospitals keen to increase preventive health in routine clinical care should undertake a mapping exercise to examine the options available to clinicians that enable preventive health interventions to occur, and what is being done well in the hospital (19). Following this, hospitals need to consult with clinicians and relevant stakeholders to explore and where possible co-design pathways to integrate preventive health recommendations into routine practice.

Clinicians ambivalent about the importance and acceptability of preventive health practice should be encouraged that patients not only appreciated the PA recommendation but welcomed it. For many participants in this study, the clinical interaction was a significant turning point toward becoming more physically active. Hospitals seeking to encourage PA promotion should consider how to implement pathways to facilitate systematic PA screening and promotion. For example, PA calculators have been incorporated into electronic medical records to ensure that a baseline PA measure is taken at the initial consultation of a pregnant woman's contact with the hospital (19). This baseline measure encourages discussions around PA and subsequent referrals to PA promotion programs (19). Clinicians seeking to encourage patients to increase PA should consider the style of communication that the patients felt influenced their decision to change, and how preventive health conversations and recommendations can be simultaneously directive toward change and supportive of patient autonomy (38).

## Limitations

There are limitations to the study. Participants in the present study had previously taken part in a study that aimed to increase PA (20). These individuals may have been more motivated to engage in PA. The purposive sampling ensured that we included participants who were allocated to both intervention and control groups, and participants who did and did not increase PA at the end of the study. The study recruited participants through an ambulatory hospital clinic in one hospital setting. This potentially restricted the diversity in participants and might limit the extension of the research findings to the population at large. Within our available sample we made a conscious effort to recruit both male and female participants across a wide age range, and represent a diversity geographic areas and levels of socio-economic disadvantage. While studies conducted from a single setting might limit transferability to all settings, the primary aim of this research was to acquire in-depth knowledge about the phenomenon studied. The steps taken to maximize study rigor and ensure data saturation (25, 26) should provide confidence in the broad applicability of the findings to other non-admitted, public hospital services.

## Conclusions

The patients in this study believed that non-admitted hospital appointments represent an opportune time to deliver health promotion initiatives, and found the delivery of recommendation to increase PA and engage in a telephone coaching program was helpful in initiating the PA behavior change process. Receiving a recommendation to increase PA by the surgeons was perceived as acceptable and influential in promoting behavior change contemplation. The provision of relevant information regarding follow-on services was also beneficial, but patients valued retaining autonomy over the decision to engage in these follow-on services, rather than being recipients of unsolicited referrals. Hospitals seeking to integrate health promotion interventions into routine care should work with clinicians to explore facilitators to increase health promotion practice, confident of the acceptability of health promotion practice to hospital patients.

## Data Availability Statement

The raw data supporting the conclusions of this article will be made available by the authors, without undue reservation.

## Ethics Statement

The studies involving human participants were reviewed and approved by Research Ethics Committees of Bendigo Health Care Group (reference number LNR/18/BHCG/44121) and La Trobe University College of Science Health and Engineering Human Ethics Sub-Committee. The patients/participants provided their written informed consent to participate in this study.

## Author Contributions

SBa, MK, and SBe conceived the project and assisted with the protocol design. SBa and KR curated that data. SBa, KR, and GB completed formal analysis. MK, SBe, and PO'H provided supervision. SBa drafted the manuscript. All authors read, edited and approved the final manuscript.

## Conflict of Interest

The authors declare that the research was conducted in the absence of any commercial or financial relationships that could be construed as a potential conflict of interest.

## Publisher's Note

All claims expressed in this article are solely those of the authors and do not necessarily represent those of their affiliated organizations, or those of the publisher, the editors and the reviewers. Any product that may be evaluated in this article, or claim that may be made by its manufacturer, is not guaranteed or endorsed by the publisher.
