# Supplementary material for: “Exercise Is My Medicine”: A Qualitative Study Exploring the Experiences of Non-admitted Patients Receiving Physical Activity Promotion From Hospital Surgeons
Source: Front Public Health. 2022 Jun 3;10:915496. doi: 10.3389/fpubh.2022.915496 (PMC9204139; doi:10.3389/fpubh.2022.915496)
Supplement: Supplementary file 1 [file Data_Sheet_1.PDF]

H4U-2 Participants experiences - Interview guide

Topics	Questions
General	Standard opening question: What was the reason you agreed to participate in the Healthy 4U-2 study?
Engaging with the surgeon	You entered the study after coming into hospital to see a surgeon- how did that happen?<br><br>*Probes: tell me more about that*<br><br>*What kind of questions did the surgeon ask? Prompt: Specifically about physical activity*<br><br>*Did the surgeon give you any advice? Prompt: Specifically about physical activity.*<br><br>*How did this conversation start?*
Receiving PA information	What are your thoughts on receiving this information from the surgeon?<br><br>*Probes: tell me more about that*<br><br>Did you expect to discuss physical activity with the surgeon?<br><br>*Probes: Why/why not?*<br><br>*How did this make you feel?*<br><br>*Do you think discussing physical activity was relevant, considering the reason you were there to see the surgeon?*<br><br>*Probes: Why/why not?*
Impact	What impact, if any, did discussing physical activity with the surgeon have on your physical activity?<br><br>*Probes: tell me more about that*<br><br>*If they say it impacted – ask for scale of impact from 1- 10y*<br><br>*Follow-up: If answer 6, ask why 6 and not 4.*<br><br>*Was there anything in particular that influenced your decision/thoughts about being more active?*<br><br>What impact, if any, did discussing physical activity with the surgeon have on your decision to enrol in the telephone coaching program?<br><br>*Probes: tell me more about that*<br><br>*If they say it impacted – ask for scale of impact from 1- 10y*<br><br>*Follow-up: If answer 6, ask why 6 and not 4.*

Surgeons discussing PA in general	What do you think about consulting surgeons discussing physical activity with patients<br><br>*Probes: Does if fit within their role as consulting surgeons*<br><br>*Why/why not?*<br><br>What do you think other people would think about consulting surgeons discussing physical activity with them during a visit?<br><br>*Probes: tell me more about that*<br><br>*Why might some people like discussing physical activity?*<br><br>*Why might some people not like discussing physical activity?*<br><br>*Can you think of any situations where surgeons defiantly should discuss physical activity?*<br><br>Have you discussed increasing physical activity with other health professionals in the past? *If yes – what other professionals*<br><br>*Any difference to the interaction with the surgeon? Tell me more…*
Overall impact	Overall, has discussing PA with the surgeon made any difference to you, and your physical activity?<br><br>If yes, what components helped with increasing physical activity?<br>*Which components were most helpful? (Prompt: in what ways?)*<br><br>*Which components were not so helpful? (Prompt: why not)*<br><br>*If not, what else might have helped?*
	Do you have anything else you would like to add, or have any comments?
